# Predicting the Wear Amount of Tire Tread Using 1D−CNN

**DOI:** 10.3390/s24216901

**Published:** 2024-10-28

**Authors:** Hyunjae Park, Junyeong Seo, Kangjun Kim, Taewung Kim

**Affiliations:** Department of Mechanical Design Engineering, Tech University of Korea, Siheung-si 15073, Gyeonggi-do, Republic of Korea

**Keywords:** tire wear prediction, 1D−CNN, bottleneck features, tire internal acceleration, tire internal pressure, tire vertical load

## Abstract

Since excessively worn tires pose a significant risk to vehicle safety, it is crucial to monitor tire wear regularly. This study aimed to verify the efficient tire wear prediction algorithm proposed in a previous modeling study, which minimizes the required input data, and use driving test data to validate the method. First, driving tests were conducted with tires at various wear levels to measure internal accelerations. The acceleration signals were then screened using empirical functions to exclude atypical data before proceeding with the machine learning process. Finally, a tire wear prediction algorithm based on a 1D−CNN with bottleneck features was developed and evaluated. The developed algorithm showed an RMSE of 5.2% (or 0.42 mm) using only the acceleration signals. When tire pressure and vertical load were included, the prediction error was reduced by 11.5%, resulting in an RMSE of 4.6%. These findings suggest that the 1D−CNN approach is an efficient method for predicting tire wear states, requiring minimal input data. Additionally, it supports the potential usefulness of the intelligent tire technology framework proposed in the modeling study.

## 1. Introduction

Excessively worn tires pose a significant risk to vehicle safety, as they drastically reduce traction, increase braking distance, and heighten the likelihood of blowouts. The traction of an excessively worn tire decreases significantly on a wet road surface [1]. This endangers the driver and passengers, as well as other road users, making the maintenance of tire tread a critical factor in preventing accidents and ensuring road safety. The replacement standard of a passenger tire typically falls in the range of 2.4 to 3.2 mm tread depth. Tires with a tread thickness of less than 1.6 mm have been found to be three times more likely to be involved in accidents [2]. Therefore, it is crucial to regularly monitor the wear condition of tires, and when they reach the wear limit, replace them. However, it is known that many drivers tend to neglect regular checks.

For this reason, research on automatically monitoring tire wear is actively being conducted. The wear prediction methods can be divided into several categories. Some researchers have proposed methods that utilize images of tire grooves. Karim et al. classified the wear state of a tire into two conditions—good or bald—using a convolution neural network (CNN) method [3]. Gürfidan et al. proposed a method to analyze the surface wear images of vehicle tires using a CNN model, achieving a classification accuracy of 99.72% [4]. Roomi et al. proposed a method to classify tire wear conditions using a transfer learning-based residual neural network (ResNet50) model [5]. Zhu et al. classified the wear state of a tire into five categories using a K-nearest neighbor (KNN) classifier, based on features extracted from images of the tire [6]. These approaches require either the tire to drive over the imaging device or cleaning of the device to obtain a clear image of the tire grooves. Such methods might be more suitable for commercial vehicles that park in specific locations than personal vehicles.

Several studies have explored monitoring tire wear by examining the correlation between tire characteristics and tread depth. While Jeong et al. demonstrated that the normalized wear amount is inversely correlated to the normalized bending stiffness of the tire, they did not validate the prediction accuracy using driving test data [7]. Singh et al. suggested that tire wear estimation can be based on data from tire inflation pressure, torsional mode frequency, and torsional mode coefficients derived from tire identification [8,9]. However, the performance metrics of this method remain undisclosed due to confidentiality restrictions. Further research by Singh et al. showed that the tire wear state can be estimated using tire internal acceleration, vertical load, and internal pressure [10]. Similarly, the performance metrics of this method were not disclosed in the literature. Wretlind et al. proposed a method to estimate the effective rolling radius by comparing GPS speed with wheel speed, using a polynomial correction model to adjust for external factors, such as speed, tire pressure, and temperature [11]. Although these concepts are promising, the change in effective rolling radius was less than 3 mm. Thus, further evaluation of this method’s performance is necessary for a wider range of tread wear amounts.

Another approach to monitoring tire wear involves the use of sensors, such as accelerometers or strain gauges, which are typically installed on the inner surface of the tires. Morinaga claimed a correlation between the deformation speed around the tire contact patch and the amount of tire wear [12]. The inventor utilized the peak tire internal accelerations and its times to calculate the deformation speed of the tire tread area. Hanatsuka claimed that the frequency content of a tire’s internal acceleration varies depending on the level of tire wear [13]. The inventor claimed that the state of the tire wear can be estimated using “frequency band value”, which is similar to dynamic stiffness obtained from a frequency spectrum of a mechanical system. Similarly, Han et al. proposed a deep neural network (DNN) algorithm to classify tire wear states by analyzing the frequency content of acceleration signals [14]. The authors extracted the average magnitudes of frequency content from the radial component of the tire acceleration for every 50 Hz interval while maintaining the tire’s vertical load and pressure at nominal conditions. The state of tire wear was then classified into 0%, 20%, 40%, and 80% using this information as features. Since the algorithm is a classifier rather than a regressor and was developed specifically for nominal tire vertical load and pressure conditions, its ability to predict tire wear states between these classes under varying load and pressure conditions requires further investigation. Zhang et al. developed a tire abrasion status prediction algorithm using manually selected features in a backpropagation neural network (BPNN) with a single hidden layer consisting of 20 neurons [15]. While this method showed promising results, it requires both accelerometers and strain gauges, as well as tire pressure and vertical load data. Additionally, the authors split the dataset into training and test sets without using a validation set, making it unclear how they determined the hyperparameters for their algorithm. Additionally, the aforementioned studies did not simultaneously consider tire internal pressure and vertical load, both hypothesized to be important factors in tire behavior.

Kim et al. proposed a tire wear prediction algorithm based on a 1D–CNN framework that utilizes only internal tire acceleration data [16]. One-dimensional CNN models are well-suited for processing sequential data, such as time-series or audio signals. By incorporating residual connections, the network can effectively learn deeper temporal features [17]. The 1D–CNN-based tire wear prediction algorithm was developed using finite element (FE) simulation results as training data rather than experimental data. Given that the method requires less information than previous studies and demonstrated strong performance (RMSE: 3.7%), it is worthwhile to verify its effectiveness with experimental data.

In summary, while various methods for predicting tire wear have been proposed, their performance remains limited due to insufficient data or confidentiality constraints. The goal of this study was to validate a previously proposed tire wear prediction algorithm using driving test data under diverse conditions, including tire internal pressure and vertical load [16]. Initially, driving tests were conducted on a proving ground with tires at various wear levels to measure internal accelerations. The acceleration signals were then processed using empirical functions to exclude atypical data before being input into the machine learning model. A revised 1D−CNN-based tire wear prediction algorithm was subsequently developed and trained. Finally, the algorithm’s performance was evaluated using an acceleration dataset from tires that were not part of the training phase.

## 2. Methods

### 2.1. Driving Test

#### 2.1.1. Tire Specimens

**Figure 1:**
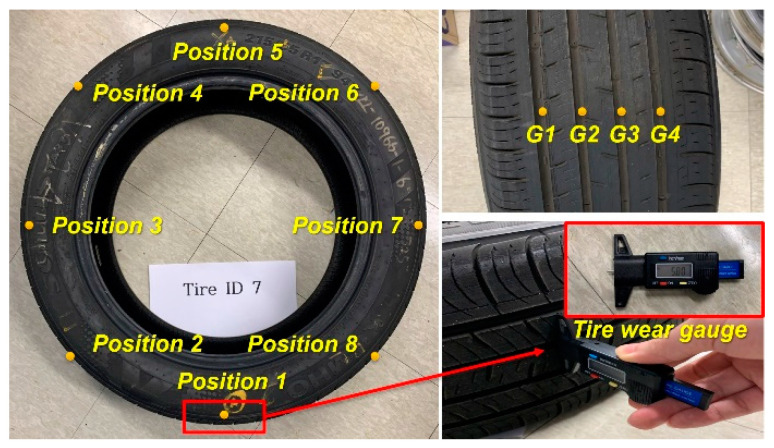
Measurement of tire wear.

The test tires used were OEM passenger tires manufactured by Kumho Tire (Gwangju, Republic of Korea), with specifications of 215/55 R17. The new tire tread depth, set by the manufacturer, was 8 mm, representing the 0% wear state (or 100% remaining tread depth). Four wear levels were considered based on this initial tread depth of (0, 2, 4, and 6) mm representing (0, 25, 50, and 75) % wear, respectively (Equation (1)). Four tires were prepared for each of these wear levels through a tire buffing process, resulting in a total of 16 tires, each assigned a unique ID (Table 1). The wear amount was determined by measuring the average tread thickness at eight points, spaced 45 degrees apart in the circumferential direction across four grooves, with an average value taken for each tire (Figure 1). Since the tires were artificially worn to approximate the target wear levels, variations in tread wear were present within the same group. For each of the four wear level groups, one tire was reserved for the test dataset, resulting in a total of four tires (ID 2, 7, 10, 14) being used to collect test data. The wear amount was then converted into a wear rate to allow quantitative comparison.

(1) $$\mathrm{Wear}=\frac{\mathrm{Current}\;\mathrm{tread}\;\mathrm{depth}\;-\;\mathrm{New}\;\mathrm{Tread}\;\mathrm{depth}}{\mathrm{New}\;\mathrm{Tread}\;\mathrm{depth}}\;[\%]$$

#### 2.1.2. Test Matrix

The driving tests considered three key parameters, namely tire internal pressure, vertical load, and vehicle speed (Table 2). Each of the 16 tires was tested under 18 different conditions. The inflation pressure conditions included three levels, which were 241 kPa (rated pressure) and ±20% of the rated pressure at (192.8 and 289.2) kPa, respectively. The load conditions involved the rated load of 340 kgf and 120% of the rated load, or 410 kgf. Driving speeds were set at (40, 60, and 80) km/h, with two round trips conducted at each speed. To ensure randomness, eight pairs of tires were randomly selected to be mounted on the left and right rear wheels of the test vehicle, and the order of the six test conditions, determined by the combinations of inflation pressure and load, was also randomized. In total, 144 tire driving tests were conducted.

#### 2.1.3. Driving Test Procedure

**Figure 2:**
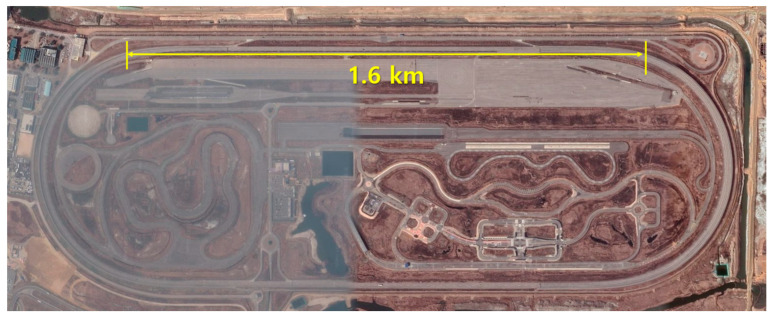
Satellite image of the test track.

**Figure 3:**
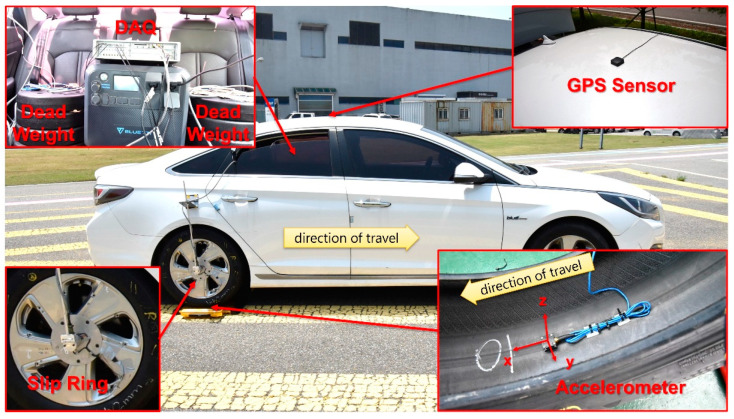
Overview of the test vehicle and instrumentation equipment.

The tire driving test was conducted on a 1.6 km long straight section of the proving ground at the Korea Automobile Testing & Research Institute (KATRI), part of the Korea Transportation Safety Authority (Figure 2). The test vehicle used was a front-wheel-drive 2016 Hyundai LF Sonata Hybrid (Figure 3). Before installing the tires on the vehicle, a 3-axis accelerometer (356A43, PCB Piezotronics, Depew, NY, USA) was attached to the centerline of the inner surface of each test tire. The x–axis of the accelerometer was aligned tangentially, perpendicular to the tire’s axis of rotation, while the z-axis pointed in the normal direction relative to the attached surface (Figure 3).

The test tires were then mounted on the left and right rear wheels of the vehicle. A GPS sensor (AA.170, Taoglas, Enniscorthy, County Wexford, Ireland) was installed on the roof to measure driving speed. Tire acceleration signals, captured by the accelerometer, were transmitted to the data acquisition system (SCADAS, Siemens, Munich, Bavaria, Germany) via a slip ring mounted on the wheel, while vehicle speed data from the GPS sensor were stored directly in the DAQ system via a wired connection. Test data were collected during the constant-speed segments of the driving tests at a sampling rate of 12,800 Hz. For each test trial, tire inflation pressure was adjusted using a vehicle air inflation device, with pressure values measured using an electronic pressure gauge (52−53097, Longacre Racing Products, Brownsburg, IN, USA). The target vertical load was verified using a portable axle scale (RWTi–200, CAS Corporation, Yangju-si, Gyeonggi-do, Republic of Korea) and adjusted by placing disk-shaped dead weights on the left and right rear seats of the vehicle.

### 2.2. Training Data Preparation

#### 2.2.1. Lowpass Filtering and Interpolation

**Figure 4:**
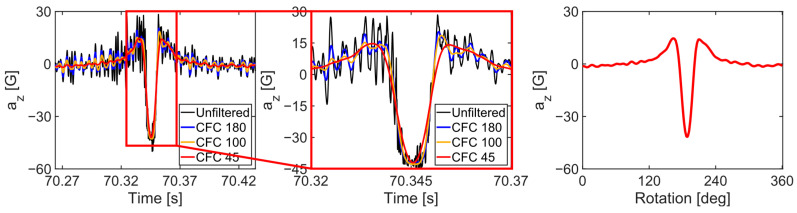
Acceleration data after lowpass filtering and interpolation.

The high-frequency components in the raw acceleration data collected from the tires were attenuated using various classes of CFC filters (SAE, 2007) (Figure 4). CFC45 was selected for tire wear prediction based on its ability to attenuate high-frequency components outside the contact region. Following the procedure outlined by Kim et al. (2023), the filtered acceleration signal for each tire revolution was mapped to the rotation angle, ensuring that each acceleration cycle started and ended at (0 and 360) degrees, respectively. The time axis was divided into 360 intervals for each cycle, and acceleration data at 361 points were obtained by linear interpolation of the filtered acceleration time history. Thus, each data point represented one degree of tire rotation.

#### 2.2.2. Screening Acceleration Dataset

**Figure 5:**
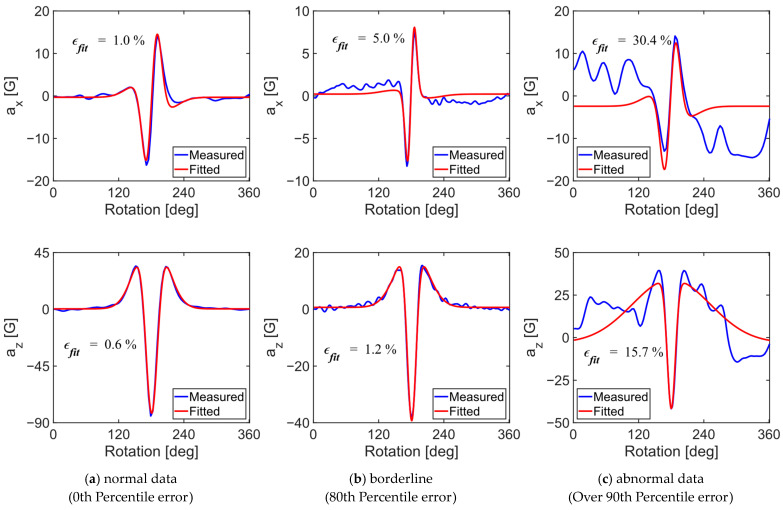
Comparison of curve-fitting errors between acceleration data and curve-fitting progress. The numbers in the figures indicate the curve-fitting error (Equation (4)).

Each acceleration cycle for normal and tangential accelerations was screened to determine whether it should be included in the dataset for the subsequent machine learning step in tire wear prediction. A curve-fitting process was employed to perform this screening objectively. Two parametric empirical functions, each consisting of two Gaussian terms, were used to fit the tangential (x-component) and normal (z-component) acceleration traces with respect to the rotation angle (Equations (2) and (3)). Kim and Kim used Equation (2) to model the shape of a bent gas turbine rotor, which closely resembled the normal component of the tire’s acceleration signal (az) (Figure 5) [18]. Furthermore, Kim et al. (2023) noted that the jerk term of the normal component of the tire’s internal acceleration was similar to that of the tangential component, leading to the derivation of Equation (3) by differentiating Equation (2).

(2) $$a_{z,fit}(\theta )=-A_{z}e^{-\alpha {\left(\theta -\theta_{s}\right)}^{2}}+B_{z}e^{-\beta {\left(\theta -\theta_{s}\right)}^{2}}+C_{z}$$

(3) $$a_{x,fit}(\theta )=A_{x}(\theta -\theta_{s})e^{-\alpha {\left(\theta -\theta_{s}\right)}^{2}}+B_{x}(\theta -\theta_{s})e^{-\beta {\left(\theta -\theta_{s}\right)}^{2}}+C_{x}$$

where

$$\begin{array}{l} \theta :tire\;rotation\\ A_{x},A_{z},\;B_{x},B_{z}\;:\;scale\;factors\\ \alpha ,\;\beta \;:\;shape\;factors\\ \theta_{s},\;C_{x},\;C_{z}:\;shift\;factors \end{array}$$

In these equations, the As and Bs served as scale factors, while α and β influenced the shape of the acceleration signal. Each acceleration trace was fitted using these equations by minimizing the normalized root mean square error (RMSE) between the lowpass filtered measured acceleration signals and the fitted function values (Equation (4)). The error rates were then sorted in ascending order, with a certain level of acceleration signals considered normal, while the remaining signals were identified as outliers and removed from the dataset for the machine learning task. The threshold for screening atypical acceleration traces was determined based on the accuracy of the tire wear prediction algorithm from the validation set.

(4) $$\varepsilon_{fit}\;[\%]=\frac{\sqrt{\sum_{i=1}^{361}{{(a}_{fit}(\theta_{i})-a_{m}(\theta_{i}))}^{2}/361}}{\max{(a}_{fit})-\min{(a}_{fit})}\times 100$$

#### 2.2.3. Separation of Training and Test Data for Tire Wear Prediction

**Figure 6:**
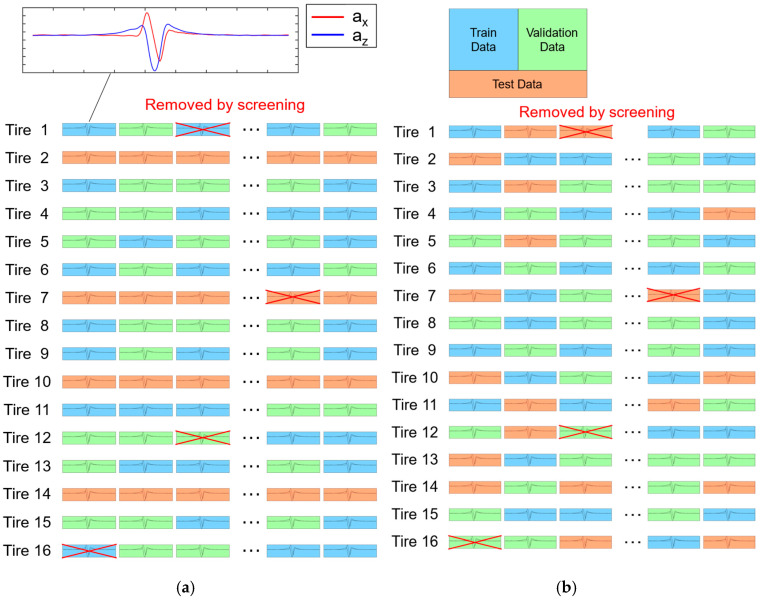
Splitting dataset into training, validation, and test sets for machine learning (**a**) by tire ID (the current study) and (**b**) randomly, regardless of tire ID (the conventional method).

The dataset for the machine learning model was created by randomly dividing the tires into training, validation, and test sets (Table 1, Figure 6). Acceleration trace data from 12 of the 16 tires were used for the training and validation phases of the tire wear prediction algorithm, while four tires (2, 7, 10, and 14) representing each wear level group were set aside for testing. The training and validation datasets were split evenly, with a 50:50 ratio. Throughout the study, 6:6:4 ratios were consistently used for training, validation, and testing to evaluate the performance of the tire wear prediction algorithms.

#### 2.2.4. Impact of Data Splitting Method on Tire Wear Prediction Accuracy

To evaluate the impact of separating the test set by tire ID, the data were also prepared using a traditional random splitting method. In this method, the 12 tires were randomly divided into training, validation, and test datasets in 45:45:10 ratios. The test data from this random split were referred to as Test 1. The data from the remaining four tires, which were completely excluded from the training and validation process, were referred to as Test 2. The performance of the tire wear prediction algorithms was then evaluated using both Test 1 and Test 2.

### 2.3. Development of Tire Wear Prediction Algorithm

#### 2.3.1. Overview

**Figure 7:**
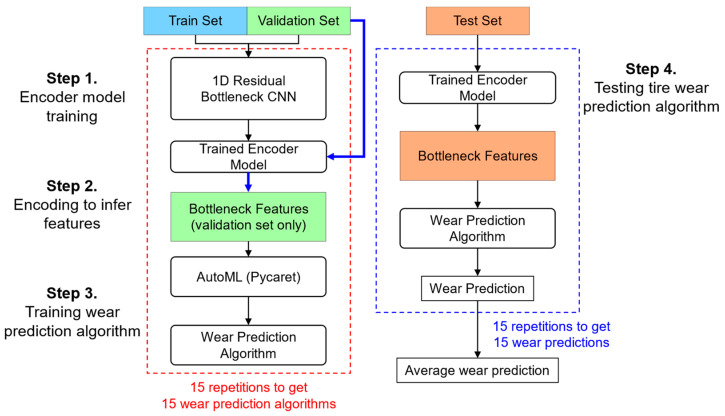
Training and evaluation process using tire acceleration data.

The training and testing of the tire wear prediction algorithms involved the following four steps: encoder model training, feature encoding, training the wear prediction algorithms, and testing the algorithm’s performance (Figure 7). First, the 1D residual bottleneck CNN algorithm was used to train the encoder model with the training data, while validation was performed using the validation data. Second, to prevent overfitting, feature encoding was carried out using only the validation data. Third, the tire wear prediction algorithm was trained using the eight encoded bottleneck features. This process was repeated 15 times to generate 15 wear prediction models, forming the basis for a simple ensemble method. Lastly, the accuracy of the tire wear prediction algorithm was evaluated using the test dataset. The following sections provide detailed descriptions of each step.

#### 2.3.2. One-Dimensional Residual CNN with Bottleneck Features

**Figure 8:**
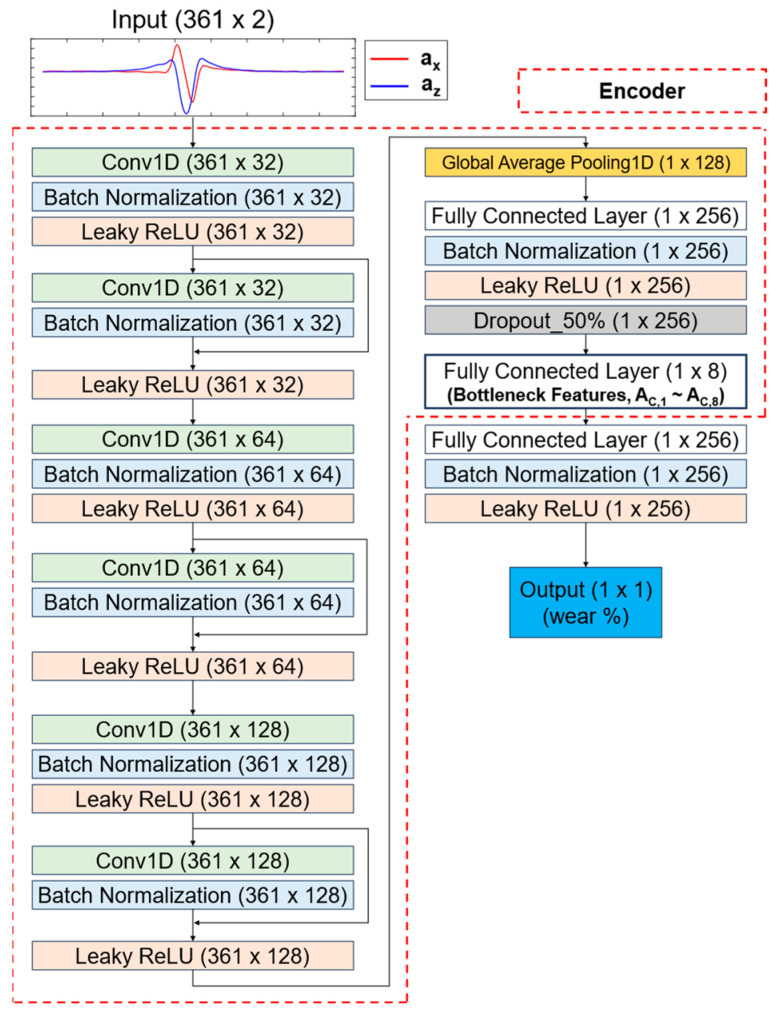
Structure of 1D residual bottleneck CNN.

In a previous study, eight bottleneck features were extracted using a one-dimensional (1D) residual bottleneck convolutional neural network (CNN) to effectively capture the key features of 1D 2-channel acceleration data [16]. In the current study, the 1D residual bottleneck CNN was designed with layers that include multiple residual connection blocks and a bottleneck layer with eight units (Figure 8). The addition of the residual layers helped mitigate the vanishing gradient problem. The 1D residual CNN, along with the bottleneck features, was trained and validated using experimental data from 12 tires (Table 1), with a 50:50 split between the training and validation sets. This process was repeated 15 times to obtain 15 trained encoder models, which were used in a simple ensemble method to predict tire wear (Step 4 in Figure 7).

#### 2.3.3. Calculation of Bottleneck Features

Fifteen separate bottleneck feature sets were calculated using the 15 trained encoder models to train the wear prediction algorithms (Figure 7 and Figure 8). Only the validation dataset was used to obtain the bottleneck features to train the tire wear prediction algorithm in the subsequent step (Step 2 in Figure 7). The decision to use only the validation set in this process was made to reduce the risk of overfitting. The training of the encoder models was kept separate from the tire wear prediction process to allow for the inclusion of additional features, such as tire pressure (P) or tire vertical load (L), together with the eight bottleneck features.

#### 2.3.4. Tire Wear Prediction Algorithm

Using the PyCaret library (version 3.0.4), artificial intelligence (AI) models were trained and employed for tire wear prediction by combining various models, excluding tree-based machine learning (ML) models [19]. This approach followed the method outlined by Kim et al. [16]. In addition to the eight bottleneck features derived from the normal and tangential acceleration data, optional inputs of the tire vertical load (L) and tire pressure (P) were included. Four feature combinations were considered for the tire wear prediction algorithms, the eight bottleneck features (Ac) alone, Ac with P, Ac with L, and Ac with both P and L. The tire wear prediction algorithms were then trained and validated using these feature sets.

#### 2.3.5. Performance Evaluation of Tire Wear Prediction

Lastly, the performance of the developed tire wear prediction algorithms was evaluated using the test data from the four tires that were excluded from the training process (Step 4 in Figure 7). The simplest ensemble model, which generates the final prediction by averaging (or voting) across multiple repetitions of the same model structure, was applied (Equations (5) and (6)).

(5) $$\varepsilon_{wear}=\sqrt{\sum_{i=1}^{N}\frac{{\left(wear_{prediction}-wear_{ground\;truth}\right)}^{2}}{N}}$$

where

(6) $$wear=\frac{current\;tread\;depth-initial\;tread\;depth}{initial\;tread\;depth}\;[\%]$$

## 3. Results

### 3.1. Threshold for Screening Acceleration Dataset

**Figure 9:**
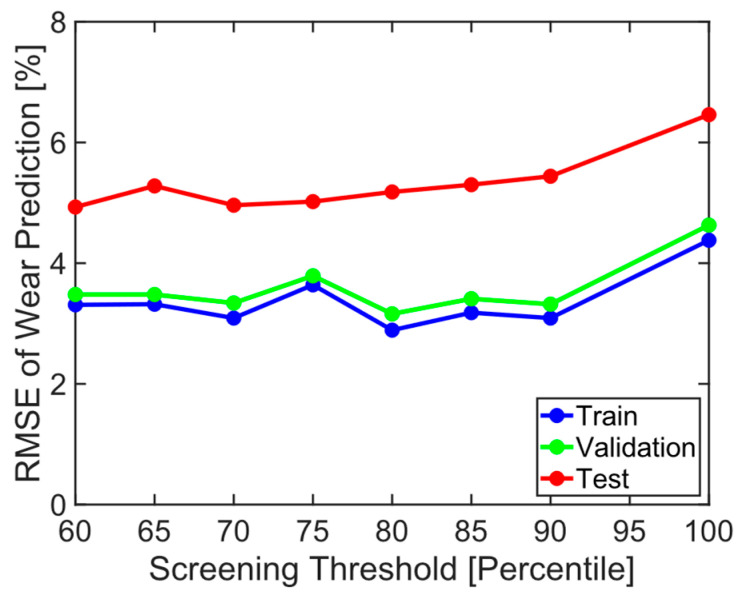
Comparison of algorithm performances by percentage of data considered normal.

Figure 9 shows three pairs of curve-fitting results for the acceleration signals, representing normal acceleration data, borderline acceleration data, and abnormal acceleration data. As the fitting error increased, the measured acceleration signals deviated from the typical shapes of the tangential (a_x_) and normal (a_z_) acceleration traces. The threshold for deciding which acceleration traces to include was determined based on the accuracy of the tire wear prediction. The prediction accuracies for the validation data were examined against various threshold values for screening acceleration data (Figure 9). The term “100%” indicates no screening, while “60%” refers to filtering out 40% of the acceleration signals with the largest errors. The performance on the test data generally improved as more filtering was applied. It was found that using the top 80% of the data yielded the best performance for the validation set. The curve-fitting error threshold for this condition was approximately 5% for ax and 1.2% for az. In other words, if the error for ax exceeded 5% or the error for az exceeded 1.2%, the data were considered abnormal and were discarded. Since both the normal and tangential acceleration signals needed to be in the top 80% simultaneously, this process screened out 73,145 acceleration cycles out of 249,192, retaining 176,047 cycles (70.64% of the entire dataset) for training, validation, and testing. Because the threshold was determined based on the validation set, this step will not be necessary for future tire wear predictions.

### 3.2. Bottleneck Features from the Encoder Model

**Figure 10:**
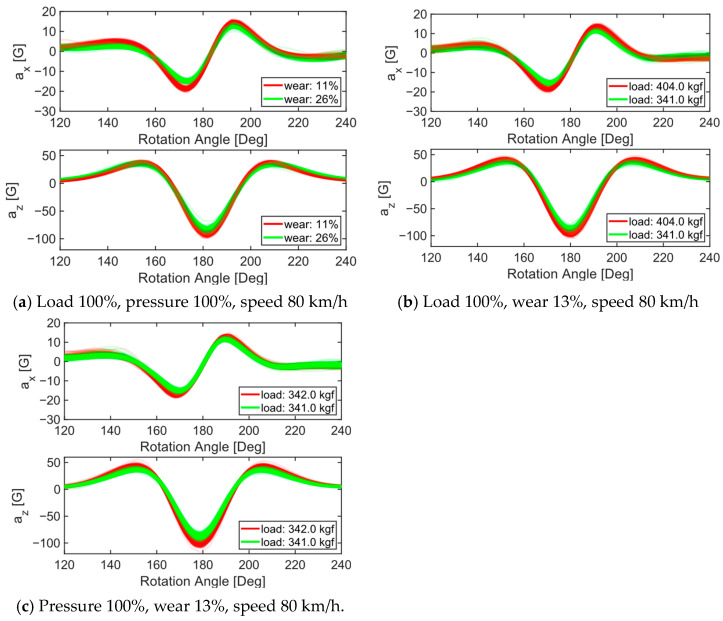
Sensitivity of tire acceleration signals with respect to (**a**) wear amount, (**b**) tire internal pressure, and (**c**) tire vertical loads.

**Figure 11:**
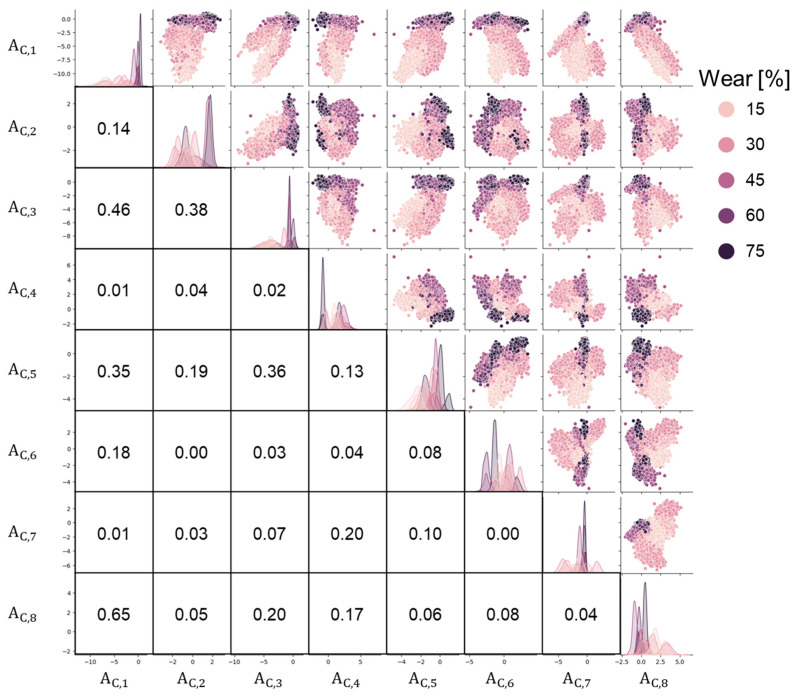
Pair plots between the eight features from the 1D−CNN with wear amount.

Figure 10 demonstrates the sensitivity of the test parameters on the tire’s internal accelerations. The pairs were selected under identical conditions, except for the differences indicated in the legends. Variability in the acceleration signals can be observed even under the same conditions, leading to overlaps between the signals from adjacent conditions. These overlaps may pose challenges for accurate wear prediction. Figure 11 shows pair plots of the eight bottleneck features (A_C,1_ to A_C,8_) obtained from the 1D−ResNet method using the training data, with wear amounts represented by five different colors. Since the training set contained over 100,000 cycles, the figure was produced by randomly sampling 1200 samples from the dataset of 12 tires (Table 1). The eight bottleneck features formed clusters corresponding to different tire wear amounts, indicating their potential usefulness to predict tire wear. For example, the cluster representing 75% wear was concentrated in the top-left corner of the scatter plot between A_C,1_ and A_C,4_. In general, A_C,1_ and A_C,2_ exhibited a monotonically increasing trend with tire wear amounts, but significant overlaps between different wear groups were observed. This overlap suggests that predicting tire wear based on a single bottleneck feature may be challenging. These eight bottleneck features were used in conjunction with AutoML for tire wear prediction.

### 3.3. Tire Wear Prediction Results

**Figure 12:**
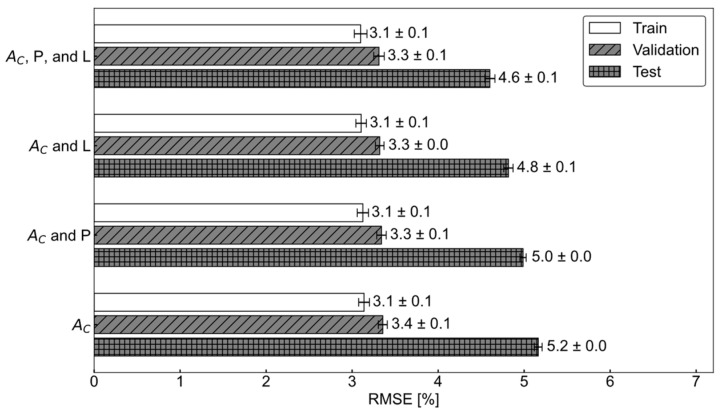
Comparison of tire wear performance with and without load and pressure information.

Tire wear amounts were predicted using four combinations of feature sets. The bottleneck features (A_C_) were used as the primary feature set, while tire internal pressure (P) and vertical load (L) were added in different combinations to evaluate their impact on prediction accuracy (Equation (5)). When only the primary features (A_C_) were used, the average prediction accuracy for the test data was 5.2%, corresponding to an average prediction error of 0.42 mm across multiple repetitions (Figure 12). Figure 12 shows an example of several trials of tire wear prediction. When tire internal pressure (P) or vertical load (L) were added as additional features, the prediction error decreased by (0.2 and 0.4)%, respectively. Interestingly, when both pressure and vertical load were included, the prediction error dropped by 0.6%, which equaled the combined individual improvements of (0.2 and 0.4)%.

### 3.4. Comparison of Data Splitting Method

**Figure 13:**
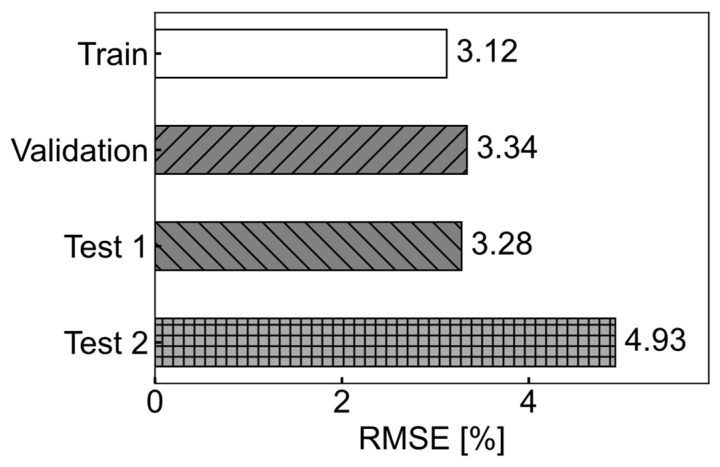
Comparisons of RMSE from training, validation, and tests.

The tire wear prediction accuracies from the two data splitting schemes—dividing the data based on tire ID versus randomly splitting the data regardless of the test specimens—were compared (Figure 13). It was found that randomly splitting the data without considering the test specimens tended to underestimate prediction errors. The RMSE of the tire wear prediction for the Test 1 dataset was 1.7% lower than that for the Test 2 dataset, which consisted of the four test tires with tire IDs of 2, 7, 10, and 14 (Table 1).

## 4. Discussion

### 4.1. One-Dimensional−CNN ResNet with Bottleneck Features

This study validated the tire wear prediction algorithm originally proposed in a previous simulation-based study by analyzing experimental data [16]. While the tire wear predictions derived from real-world measurements slightly underperformed compared to the simulation-based results, the findings highlight the utility of computational models as powerful tools to advance smart tire system development in practical applications (Figure 12). Similar approaches have been taken by Behroozinia et al. [20] and Li et al. [21] with notable success.

The current study demonstrated that bottleneck features (A_C_) extracted from a 1D−CNN effectively predicted tire wear using internal tire acceleration data (Figure 12 and Figure 13). The 1D–CNN method was chosen for its ability to process sequential data, such as time-series or audio signals. One-dimensional–CNNs allow for the easy extraction of features at various scales, making them advantageous for capturing diverse information within data. They are widely used in various applications, including personalized biomedical data classification, structural health monitoring, anomaly detection in power electronics, and electrical motor fault detection [22]. One of the advantages of the 1D–CNN approach is that it eliminates the need for manual feature extraction from time-series data. In one study, a motor fault detection algorithm was proposed based on motor current time histories using 1D–CNN [23]. Additionally, another study employed 1D–CNNs to recognize music emotions [17].

The bottleneck structure was designed to automatically extract and compress information from the normal and tangential acceleration data [24]. Bottleneck features are known for their ability to capture essential information from input data, which enhances model interpretability while improving generalization performance through lower-dimensional data representations. For example, one study used a bottleneck structure in a 1D–CNN to effectively compress relevant information while discarding irrelevant features, significantly improving model performance [25]. In that study, the authors achieved high accuracy (98.29%) and sensitivity (97.42%) in detecting seizures from EEG signals. Similarly, another study also employed bottleneck features to align the network’s data learning performance more closely with human capabilities [26].

Additionally, residual connections were introduced in the current study to address the vanishing gradient problem commonly found in deep networks. By adding the input and output of each convolution block, residual connections enable efficient training even as the network deepens, preventing performance degradation [27]. Incorporating residual connections in 1D–CNNs helps mitigate issues such as gradient vanishing in deeper networks, resulting in enhanced model performance [28].

Lastly, hyperparameter tuning was efficiently carried out using PyCaret (version 3.0.4), which automates the process of optimizing model performance [19]. Leveraging PyCaret’s built-in hyperparameter optimization capabilities allowed the study to quickly identify the best combination of parameters, significantly enhancing model performance while reducing manual effort.

### 4.2. Performance of Tire Wear Prediction Algorithm

**Figure 14:**
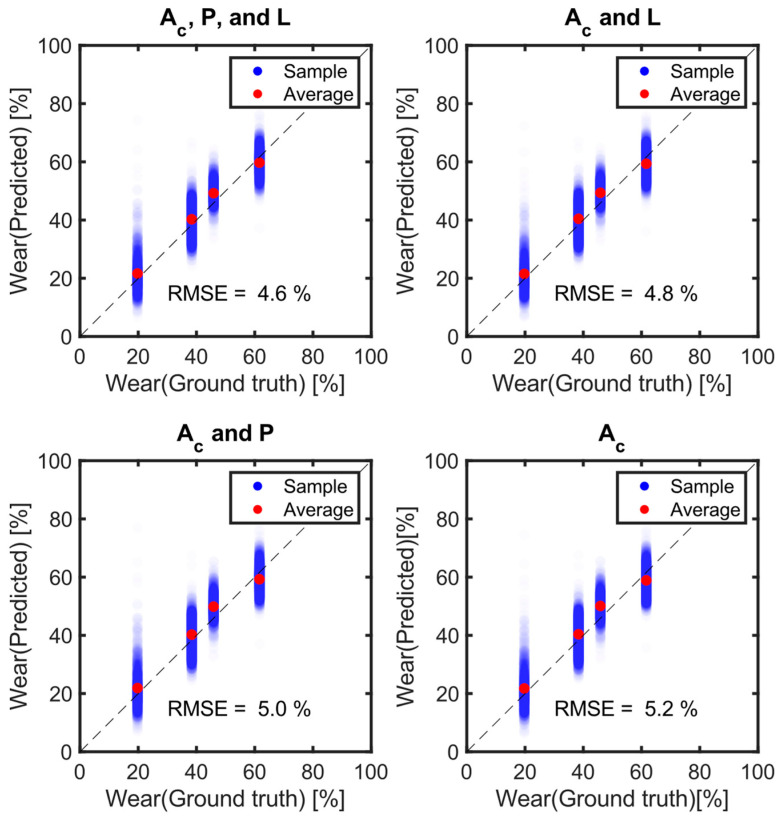
Scatter plots between the true wear and the predicted wear amounts.

The proposed algorithm, which exclusively utilizes A_C_ information, achieved an average RMSE of 5.2% (equivalent to 0.42 mm), which was higher than the accuracy of the previous modeling study (3.7%). The discrepancy between the experimental and modeling data can likely be attributed to several factors, including variations in road conditions, vehicle dynamics, and tire-to-tire differences. Unlike the idealized perfectly flat road surface used in the simulation model, real-world conditions rarely offer such uniformity. During the driving tests, vertical vibrations caused by road irregularities or tire imperfections may have introduced fluctuations in the vertical load on the tires, thereby affecting the internal acceleration signals. The fluctuation in vertical load can produce changes in the tire’s internal acceleration signals similar to those caused by tire wear, which may pose challenges for accurate wear prediction (Figure 10). Notably, only the static vertical load was used as a feature, rather than dynamic vertical loads, in training the tire wear prediction algorithm (Figure 14). Therefore, incorporating dynamic vertical load-related quantities could potentially improve the accuracy of the tire wear prediction. Additionally, the tires in this study were artificially worn through a buffing process, which is a common step in tire retreading. This process, along with inherent tire-to-tire variability from manufacturing, likely contributed to further deviations. In the simulation, tread depth was uniformly reduced across the tire model to simulate wear, whereas the actual tire specimens exhibited variations in tread depth across the eight measurement locations, potentially leading to oscillations that affected the internal acceleration signals. Lastly, the simplest ensemble method (RMSE: 5.4%) produced a 10% lower prediction error than the average error of the 15 individual models (RMSE: 5.8%), even outperforming the best model, which had an RMSE of 5.5%.

### 4.3. Data Screening Based on Empirical Functions

To address the variability in tire acceleration signals under the same test conditions—defined by tire wear amount, vehicle speed, vertical load, and internal pressure, the quality of the acceleration signals was evaluated using curve fitting to screen out inconsistent data. Empirical functions, modeled as a linear combination of two Gaussian functions, were applied to the signals (Equation (1)) [18]. The threshold for screening, treated as a hyperparameter, was determined based on validation accuracy. A significant improvement in prediction accuracy was observed when 10% of the acceleration data was excluded (Figure 9), with only marginal gains up to a 20% exclusion. It is important to note that the fitted function was used solely for screening, while the actual acceleration signals were retained for training the tire wear prediction algorithm.

### 4.4. Various Sensing Options

It was found that tire wear can be predicted sufficiently well using only the bottleneck features derived from internal tire acceleration data without the need for pressure and load information. This result aligns with the findings of Kim et al., who conducted a similar analysis using acceleration data generated from finite element (FE) analyses [16]. Although pressure and load clearly affect tire deflection, their inclusion only marginally improved wear prediction performance. Adding either tire internal pressure, vertical load, or both reduced the prediction error by less than 0.7% RMSE (an 11% improvement compared to using only A_C_ data). Tire wear prediction relied primarily on the shape of the tire’s internal acceleration signal.

The ability to predict tire wear solely based on internal acceleration is consistent with the previous modeling study [16]. As tire acceleration was analyzed in relation to the tire’s rotation angle, the signal shape was influenced by factors such as vertical stiffness, tread depth (which affects tire stiffness), angular speed, vertical load, internal pressure, and more [29]. Since a 20% change in either tire pressure or vertical load can significantly alter the tire’s signal shape, it was expected that including these variables would improve the accuracy of tire wear prediction (Table 2). However, incorporating tire pressure and vertical load as features only reduced the prediction error by a maximum of 0.7% RMSE. In a previous study (Figure 12), it was hypothesized that pressure and load information might already be encapsulated within the bottleneck features [16].

### 4.5. Data Splitting Method

Two methods of splitting the training, validation, and test data were considered, and the way the data were split had a significant impact on the performance of the tire wear prediction algorithms (Figure 13). This finding contrasts with results from a simulation-based study, where the tire wear prediction accuracy was largely insensitive to different data splitting schemes [16]. This discrepancy may be due to the simulation data being obtained under ideal conditions, where no variations existed between the tires except for the wear amount. Additionally, the simulation was conducted on a perfectly flat surface without a vehicle. In contrast, with experimental data, the machine learning algorithm may capture specific characteristics of each tire specimen rather than the general trend in tire response changes to wear. As a result, it is recommended that to improve its generalizability when developing a tire wear prediction algorithm, tire specimens be separated for training/validation and testing. While it is not always explicitly stated how datasets are split, some previous studies on tire wear prediction algorithms might have faced similar issues with data splitting [14,15]. Based on these publications, most studies tested only one tire for each wear amount of interest, suggesting that the datasets may not have been split by tire specimen.

### 4.6. Limitations

The current study has several limitations worth noting. First, the current findings are based on experimental data collected under well-controlled driving conditions. Additionally, the tire specimens in this study were artificially worn down through a buffing process, which may not fully replicate tread conditions found in real-world scenarios. Therefore, the proposed algorithm needs further validation and improvement using real-world data. Second, while the acceleration data in this study were gathered using wired systems, real-world applications will require wireless acceleration measurement systems. However, accelerometer signals from wireless systems often contain more noise and may have reduced accuracy. Third, the proposed algorithm was developed using acceleration signals obtained from a single OEM (original equipment manufacturer) passenger tire model. Therefore, it would be meaningful to investigate whether the proposed algorithm would work for different tire models through a transfer learning method. Lastly, the performance of the proposed algorithm was not compared with previous studies due to differences in the types of algorithms (regression vs. classification) [14], variations in test parameters (such as pressure or load) [15], and the lack of detailed performance information [10].

## 5. Conclusions

This study validated the effectiveness of the 1D−CNN approach in predicting tire wear using experimental data. Initially developed from tire rolling simulations based on a validated FE tire model, this method has shown potential for advancing smart tire technologies. The bottleneck features extracted from the tire’s internal acceleration using the 1D−CNN predicted tire wear with reasonable accuracy. Additionally, incorporating features such as tire internal pressure or vertical load further improved prediction accuracy. However, as these findings are based on well-controlled driving conditions, the proposed method still requires validation with real-world driving data.

## Figures and Tables

**Table 1:** Tread wear of test tires.

Tire ID	Tread Wear Average [mm]	Tread Wear Average [%]	Purpose
1	1.0	12.5	training, validation
2	1.6	19.8	test
3	1.6	19.5	training, validation
4	0.9	11.0	training, validation
5	2.3	28.8	training, validation
6	2.4	30.4	training, validation
7	3.1	38.4	test
8	2.0	25.5	training, validation
9	4.3	53.4	training, validation
10	3.7	45.9	test
11	4.0	50.6	training, validation
12	4.0	50.3	training, validation
13	6.1	76.5	training, validation
14	4.9	61.6	test
15	5.2	64.4	training, validation
16	5.9	74.0	training, validation

**Table 2:** Test matrix for tire driving tests.

Test Parameter [Unit]	Values	Number of Test Conditions
Pressure [kPa]	193, 241, 289	18
Vertical Load [kgf]	340, 410	
Velocity [km/h]	40, 60, 80	

## Data Availability

Data may be available upon request to interested researchers.
